# Supplementary material for: WordCommentsAnalyzer: A windows software tool for qualitative research
Source: F1000Res. 2018 Sep 4;7:536. Originally published 2018 May 3. [Version 2] doi: 10.12688/f1000research.14819.2 (PMC6137411; doi:10.12688/f1000research.14819.2)
Supplement: Supplementary file 3 [file f1000research-7-17614-s0002.tgz › b2a79496-4ada-47c4-9682-a99ce867836d.zip!/Eight Iranian Health Journals/The Journal of Toloo-e-Behdasht_9.docx]

© The Journal of Toloo-e-Behdasht. This work is licensed under CC BY 4.0 (https://creativecommons.org/licenses/by/4.0/).

Volume & Issue: دوره 15 - شماره 5

Title: بررسی آلودگی باکتریایی کبدهای عرضه شده در قصابی های شهر یزد در سال 1393

http://tbj.ssu.ac.ir/article-1-1176-fa.html

این مقاله حاصل پایان نامه دانشجویی کارشناسی ارشد گروه بهداشت و ایمنی مواد غذایی در دانشکده بهداشت دانشگاه علوم پزشکی شهید صدوقی یزد است. مقدمه: بیماری های باکتریایی منتقله از مواد غذائی در اکثر نقاط جهان، خسارات بهداشتی و اقتصادی زیادی را فراهم می آورد. در ایران مصرف جگر خام یا کم پخته بر اساس عادات غذایی نادرست و باورهای غلط وجود دارد که زمینه ساز انتقال عوامل باکتریایی از این طریق است. لذا هدف از این مطالعه تعیین میزان آلودگی باکتریایی جگر های عرضه شده در قصابی های شهر یزد در سال 1393 بود. روش بررسی: در این مطالعه توصیفی- مقطعی تعداد 60  نمونه جگر از قصابی های موجود در سطح شهر یزد به صورت تصادفی جمع آوری گردید. نمونه ها از نظر شمارش کلی باکتریایی، شمارش اشریشیا کلی،  شمارش استرپتوکوک های مدفوعی و  وجود یا عدم وجود سالمونلا  مورد آزمون میکروبی قرار گرفتند. داده ها با استفاده از آزمون های آماری توصیفی و نرم افزار SPSS نسخه 18 تجزیه و تحلیل گردید. یافته ها: از مجموع 60 جگر که مورد آزمون میکروبی قرار گرفتندکلیه نمونه ها(100%) آلودگی به اشریشیا کلی را نشان دادند، همچنین تعداد 4 نمونه ( 67/6 درصد) آلوده به استرپتوکوک های  مدفوعی و تعداد 4  نمونه ( 67/6 د رصد) نیز آلود ه به سالمونلا بود. نتیجه گیری:  با توجه به آلودگی باکتریایی قابل توجه به باکتری های پاتوژن روده ای و مصرف خام و نیم پخته جگر های عرضه شده در بین مردم، توجه بیشتر به رعایت اصول بهداشتی در مراحل کشتار و عرضه این محصول جهت جلوگیری از وقوع بیماری های منتقله از غذا ضرورت دارد.

Title: بررسی تولید ورمی کمپوست از باگاس اصلاح شده و پسماند آشپزخانه با استفاده از کرم ایزنیا فوئیتیدا

http://tbj.ssu.ac.ir/article-1-1257-fa.html

مقدمه: رشد جمعیت و شهرسازی، مدیریت مواد زائد جامد را به یکی از بزرگترین چالش های زیست محیطی در دنیا تبدیل کرده است و در سالهای اخیر استفاده از کرم های خاکی برای دفع زائدات و تبدیل آنها به مواد سودمند مورد توجه قرار گرفته است.هدف از این مطالعه تولید ورمی کمپوست از باگاس و پسماندهای آشپزخانه توسط کرم خاکی ایزنیا فوئیتیدا می باشد. روش بررسی: در این مطالعه ک بصورت تجربی انجام شد، تیمارهای باگاس و مواد زائد آشپزخانه با سه بار تکرار ( در مجموع 9 واحد آزمایشگاهی) تهیه شد. پارامترهایpH ، کل کربن آلی، نیتروژن کجلدال، فسفر دردسترس و پتاسیم کل به مدت 60 روز اندازه گیری شد. یافته ها: نتایج نشان داد که با گذشت زمان  pHفرایند روند کاهشی داشته، و در تیمار شاهد مقدار 13/0±36/6، در نسبت 1:1 (مخلوط باگاس با زائدات آشپزخانه)15/0±79/6و در تیمار با نسبت 1:2 62/2±58/6 حاصل شد. .همچنین نتایج کاهش در کل کربن آلی (TOC) ،نیتروژن کجلدال (TKN) ، C/N و افزایش در فسفردر دسترس(P) و پتاسیم (K) را نشان داد. در تیمار شاهد مقادیر TOC، TKN و C/N به ترتیب 41/20%، 5/9% و 12% کاهش یافته و مقادیر P و K 32% و 37/31% افزایش یافت. در نسبت 1:1 مقادیر TOC، TKN و C/N به ترتیب 4/50%، 7/39% و9/17% کاهش یافته و مقادیر P و K 5/50% و 7/54% افزایش یافت. در نسبت 1:2 مقادیر TOC، TKN و C/N به ترتیب 54%، 33% و 2/31% کاهش یافته و مقادیر P و K 18/52% و 17/57% افزایش یافت. نتیجه گیری: مناسبترین مخلوط کمپوست باگاس با زائدات آشپزخانه جهت تهیه ورمی کمپوست در نسبت 1 به  2 مشاهده شد و در اکثر پارامترهای های اندازه گیری شده برتری خود را نسبت به سایر تیمارهای مورد آزمایش آشکار نشان داد.

Title: بررسی میزان بنزن ، تولوئن ، اتیل بنزن و زایلن (BTEX ) موجود در هوای محیطی کارگاههای نقاشی خودرو در مقایسه با هوای مناطق مسکونی اطراف کارگاهها در شهر یزد - زمستان 1394

http://tbj.ssu.ac.ir/article-1-2124-fa.html

مقدمه: بنزن، تولوئن، اتیل بنزن و زایلن (BTEX) از جمله ترکیبات آلی فرار و آلاینده های مهم هوا می باشند که خصوصیات فیزیکی و شیمیایی مشابهی دارند. این ترکیبات از حلال ها ، مواد پتروشیمی و رنگ ها به وفور متصاعد می‌گردند و در مقادیر جزئی نیز مخاطراتی برای شاغلین صنایع و همچنین مناطق مسکونی اطراف آنها بهمراه دارند. هدف از انجام این مطالعه بررسی و تعیین میزان این آلاینده ها درون هوای محیطی کارگاه های نقاشی خودرو و همچنین مناطق مسکونی اطراف کارگاه های شهر یزد می باشد. مواد و روش ها:تعداد 15 نمونه از هوای محیطی کارگاه‌های نقاشی خودروی شهر یزد وتعداد 15 نمونه نیز از هوای مناطق مسکونی اطراف آن کارگاه‌ها در فصل زمستان سال 1394 توسط کیسه‌های تدلار و پمپ نمونه برداری فردی جمع آوری گردید. نمونه‌ها با فاز جامد میکرو استخراج شده و با استفاده از گازکروماتوگرافی (GC/FID) جداسازی و سنجش گردیدند. داده ها با نرم افزار آماری16 SPSS- مورد تجزیه و تحلیل قرار گرفتند. یافته‌ها: نتایج حاصل نشان دادکه میانگین غلظت و انحراف معیار هر یک از آلاینده های بنزن، تولوئن،                  اتیل بنـزن و زایـلن در هوای محیطـی کارگاهـهای نقاشـی خودروی شهر یزد به ترتیب (73/0± 14/2)،                  ( 97/1± 09/4 ) ، ( 290/0± 08/1 ) و( 00/1 ± 05/3) ppm و میانگین غلظت و انحراف معیار هر یک از آلاینده های بنزن، تولوئن، اتیل بنزن و زایلن در هوای شعاع 50 متری مناطق مسکونی اطراف کارگاهها به ترتیب ( 202/0 ± 960/0 ) ، ( 409/0± 50/1 ) ، ( 420/0± 613/0 ) و ( 452/0 ± 38/1 ) ppm می باشد. نتیجه گیری: میانگین غلظت بنزن در هوای کارگاههای نقاشی خودرو چند برابر بیشتر از میزان حد مجاز استاندارد بیان شده توسط اداره ارزیابی خطرات بهداشت محیط و کمیته فنی بهداشت حرفه ای کشور (5/0) ppm می باشد و همچنین میانگین غلظت این آلاینده در هوای شعاع  50 متری اطراف کارگاهها نیز بیشتر از میزان مجاز استاندارد می باشد در حالیکه غلظت تولوئن ، اتیل بنزن و زایلن کمتر از حد مجاز می باشند

Title: بررسی میزان تاثیر آموزش بر رفتارهای پیشگیری کننده سرطان پرستات در مردان بالای 40 سال شهر یزد بر مبنای مدل اعتقاد بهداشتی

http://tbj.ssu.ac.ir/article-1-1282-fa.html

مقدمه: سرطان پروستات شایع ترین سرطان بین مردان می باشد، بر همین اساس هدف از مطالعه حاضر تعیین میزان تاثیر آموزش بر رفتارهای پیشگیری کننده از سرطان پروستات در مردان بالای 40 سال شهر یزد بر مبنای مدل اعتقاد بهداشتی می باشد. روش بررسی: این مطالعه یک مطالعه تجربی- مداخله ای می باشد، حجم نمونه های  گروه های مورد و شاهد بر اساس فرمول 40 نفر بدست آمد و سپس نمره مربوط به نگرش و آگاهی و عملکرد افراد نمونه قبل از آموزش و سپس 3 ماه بعد از مداخله بر اساس پرسشنامه  بدست آمد و اطلاعات بدست آمده وارد نرم افزار18  SPSS شده و به کمک آزمون های تی مستقل و کای اسکوئر بررسی شد . یافته ها: در این مطالعه میانگین سنی افراد شرکت کننده در گروه آزمون 33/8 ±55/54 و میانگین سنی افراد شرکت کننده در گروه کنترل 69/8±02/55 می باشد،. مقایسه نمره آگاهی در گروههای آزمون و کنترل قبل و بعد از مداخله نشان داد در مرحله دوم (بعد از مداخله) اختلاف میانگین نمره آگاهی در گروه آزمون و کنترل معنادار بود(000/0 =P)بین سازه شدت درک شده با موانع درک شده و منافع درک شده ، با خود کارآمدی (000/0 = P) عملکرد (000/0 = P) راهنمای عمل (000/0 = P) همبستگی مستقیم معناداری مشاهده شد.  نتیجه گیری: این مطالعه نشان داد که با افزایش آگاهی در بین افراد بالای 40 سال می توان عملکرد های پیشگیری از بروز سرطان پروستات را بهتر و موثر تر در جامعه به کار برد.

Title: بررسی آگاهی، نگرش و عملکرد زنان مراجعه کننده به مراکز بهداشتی شهر یزد درمورد مواد غذایی آلوده به سم آفلاتوکسین

http://tbj.ssu.ac.ir/article-1-1420-fa.html

چکیده مقدمه:آفلاتوکسین ها متابولیت های سمی هستند که بوسیله قارچ های معینی در خوراک انسان و دام تولید  می شوند. با صدمه زدن به DNA می تواند باعث ناقص الخلقه زایی و همچنین سرطانزایی و سرطان کبد  شود. این تحقیق با هدف بررسی آگاهی، نگرش و عملکرد زنان شهر یزد نسبت به مواد غذایی کپک زده و آلوده به سم افلاتوکسین صورت گرفته است. روش بررسی: این مطالعه توصیفی در سال 1393 بر روی 152 زن مراجعه کننده به مراکز بهداشتی شهر یزد صورت گرفت. نمونه گیری به صورت چند مرحله ای انجام شد. اطلاعات به وسیله  پرسشنامه محقق ساخته جمع آوری گردید. روایی پرسشنامه از طریق روایی محتوا و پایایی آن به روش پایایی هم ارز تعیین گردید. داده ها پس از گردآوری با استفاده از نرم افزار SPSS (نسخه 20) مورد تجزیه و تحلیل قرار گرفت. یافته ها: 107 نفر (70 %) از افراد مورد مطالعه  نسبت به آفلاتوکسین آگاهی ضعیف و تنها 6/2 درصد شرکت کنندگان دارای اطلاعات نسبتا خوبی درمورد سم آفلاتوکسین بودند. در بررسی کلی سطح عملکرد  38 نفر (25%) عملکرد خوب، 108 نفر (1/71%) عملکرد متوسط و 6 نفر (9/3%) عملکرد ضعیف داشتند. 25% ، 3/74% و 7/0% از شرکت کنندگان به ترتیب نسبت به عوارض و مخاطرات مواد غذایی کپک زده و آلوده به آفلاتوکسین دارای نگرش مثبت، متوسط و منفی بودند. نتیجه گیری:با توجه به یافته های پژوهش، آگاهی ضعیف شرکت کنندگان نسبت به آفلاتوکسین و متوسط بودن سطح نگرش و عملکرد اکثر شرکت کنندگان ارایه راهکارهایی جهت افزایش سطح آگاهی افراد  در راستای افزایش سطح نگرش و عملکرد آنها و کاهش مواجهه با سم آفلاتوکسین لازم و ضروری بنظر می رسد.

Title: بررسی میزان آگاهی ، نگرش و عملکرد خانوارهای پرمصرف شهر یزد نسبت به مصرف صحیح آب

http://tbj.ssu.ac.ir/article-1-1213-fa.html

مقدمه: رشد روز افزون جمعیت، توسعه شهرنشینی ناشی از مهاجرت به شهرها و نیز افزایش تقاضا از مولفه هایی هستند که حجم آب در دسترس را تحت تاثیر قرار می دهند. لذا صرفه جویی در مصرف آب در تمام شهرها و بخصوص یزد از اهمیت بسیاری برخوردار است.  روش بررسی: این تحقیق به صورت توصیفی- مقطعی و بر روی خانواده های پرمصرف انجام شد. نمونه گیری به صورت خوشه ای و در دو مرحله انجام شد و در نهایت 188 پرسشنامه توسط نرم افزار آماری SPSS(T-test ,  Mann-Whitney) تجزیه و تحلیل شد. یافته ها: میانگین نمرات آگاهی  15/5±04/60 ، نگرش 97/3±28/22 و عملکرد 63/3 ± 6/11 بدست آمد. این مطالعه نشان داد آگاهی کل ارتباط معنی داری با شغل و جنس دارد. بر اساس نتایج تحلیل عاملی نیز مشخص شدکه حیطه "سایر بخش های منزل(کولر، شستشوی اتومبیل، وسایل کاهنده مصرف آب و...)" بیشترین و حیطه" علل هدر رفت آب در منزل (شستشوی پله ها، نشت آب از لوله ها، بازگذاشتن شیر آب هنگام مسواک زدن و...) " کمترین سهم را در بیان آگاهی کل داشتند. نگرش و عملکرد افراد در این مطالعه با هیچ کدام از متغیر های مورد بررسی ارتباط معنی داری را نشان نداد.  نتیجه گیری: نتایج مطالعه حاضر نشان داد که خانوارهای مورد مطالعه دارای سطح نسبتاً خوبی از عملکرد نسبت به رفتارهای صرفه جویی در مصرف آب بودند ولی تلاش های مداخله ای در راستای ارتقاء این رفتارها به سطح عالی، مهم و ضروری می باشد.

Title: بررسی آگاهی، نگرش و عملکرد مادرانِ دانش آموزان دختر پایه هفتم و سوم راهنمایی شهر زنجان در مورد تغییرات روانی دوران بلوغ و ارتباط آن با روابط بین نوجوانان و مادران

http://tbj.ssu.ac.ir/article-1-1009-fa.html

مقدمه: دوران بلوغ  از اهمیت زیادی برخودار بوده و تغییرات جسمی-روانی و اجتماعی در این دوران رخ           می دهد. این دوران در زندگی بزرگسالی و نیز آینده جامعه تأثیر زیادی دارد وبا توجه به افزایش کشمکش های بین والدین و فرزندان در این دوران و نقش والدین و بخصوص مادر به دلیل ساخت خانواده ها در کشور ما در پایه گذاری شخصیت سالم فرزندان، این تحقیق با هدف بررسی میزان  آگاهی، نگرش و عملکرد مادران در مورد تغییرات روانی دوران بلوغ و ارتباط آن با روابط بین دختران و مادران انجام شد. روش بررسی:در این مطالعه توصیفی، تعداد 161 دانش آموز دختر مقطع دوره اول متوسطه (74 نفر پایه هفتم و 87 نفر پایه سوم راهنمایی) شهر زنجان که به صورت نمونه گیری تصادفی چند مرحله ای از شش مدرسه مقطع دوره اول متوسطه انتخاب شده بودند، وارد مطالعه شدند. وسیله جمع آوری داده های مربوط به آگاهی، نگرش و عملکرد مادران  یک پرسشنامه محقق ساخته  شامل 18 سؤال آگاهی، 20سؤال نگرشی و20 سؤال عملکردی بود. وسیله جمع آوری داده های مربوط به رابطه دختر و مادر، پرسشنامه استاندارد ارزیابی رابطه ولی- فرزندی بود که 24 سؤال داشت وتوسط دانش آموزان تکمیل گردید.داده ها پس از جمع آوری توسط نرم افزار SPSS-18  و آزمون های آماری آنالیز واریانس و رگرسیون و همبستگی در سطح معنی داری 05/0=α مورد تجزیه و تحلیل قرار گرفت. یافته ها: میانگین نمره آگاهی مادران در مورد تغییرات غیر جسمی بلوغ 48/3±58/9 از 18 نمره ومیانگین نمره نگرش آن ها 84/8±81/64 از 100نمره و میانگین نمره عملکردشان، 82/5±4/57 از 80 نمره بودکه هر سه در محدوده متوسط قرار داشتند. میانگین نمره کل رابطه فرزند و مادر، برحسب جمع میانگین زیر مقیاس ها، 44/20 بود که در محدوده رابطه متوسط قرار داشت. رابطه فرزند و مادر با نگرش و عملکرد مادر همبستگی داشت. نگرش مادر نیز با عملکرد وی همبستگی داشت. نتیجه گیری: آگاهی بیشتر مادران در مورد بلوغ در سطح متوسط و پایین بود و بیشتر آنها نگرش مثبت وعملکرد متوسطی داشتند و با فرزندان نوجوان خود رابطه متوسطی برقرار می کردند. رابطه فرزند و مادر با نگرش و عملکرد مادر همبستگی داشت. بنابر این به نظر می رسد که با افزایش آگاهی مادران در مورد تغییر و تحولات دوران بلوغ و ایجاد نگرش مثبت نسبت به تغییرات و انجام اقدامات لازم در این زمینه و به تبع آن عملکرد خوب آنها، می تواند در برقراری ارتباط مناسب بین دانش آموزان نوجوان و مادرانشان مؤثر باشد.

Title: بررسی دیدگاه زنان و مردان تک فرزند خوزستانی درباره فرزندآوری :یک پژوهش کیفی

http://tbj.ssu.ac.ir/article-1-1653-fa.html

چکیده مقدمه: در سال های اخیر، تحولات جمعیت شناختی چشمگیری در دنیا رخ داده است.یکی از مهم ترین این تغییرات، کاهش بی سابقه باروری در تمام مناطق دنیا بوده است. به موازات این تحولات، جمهوری اسلامی ایران نیز تغییرات گسترد ه ای را تجربه کرده، به طوریکه طی سه دهه گذشته، میزان باروری در ایران به طرز حیرت انگیزی کاهش یافته است. هدف از مطالعه حاضرتفسیر دیدگاه زنان و مردان تک فرزند خوزستانی درباره فرزندآوری بود تابه این پرسش اصلی که کدامیک از عوامل ،تاثیرگذار بر امر گرایش به فرزندآوری در استان خوزستان است، پاسخ مناسب دهد. روش:مشارکت کنندگان در این مطالعه شامل 35 نفر از جمعیت زنان و مردان در محدوده سنی 45-15 سال بودند که بطور هدفمند از مراکز بهداشت و درمان شرق و غرب اهواز در سال 94-1393 انتخاب شده بودند.داده های بدست آمده به روش تجزیه و تحلیل محتوا (Thematic Analysis ) تحلیل شد. یافته ها :دو طبقه عمده شامل مولفه هایی که باعث سوق دادن زوجین به فرزندآوری(قومیت، عزت نفس والدین، احساس مثمر ثمر بودن) و مولفه هایی که مانع سوق دادن زوجین به فرزندآوری(اقتصاد،شغل والدین،وظیفه وبیماری والدین) می شوند ارایه گردید. نتیجه گیری: نتایج این پژوهش ، تجربیات زوجین از فرزندآوری بود.تقویت عواملی که باعث سوق دادن زوجین به فرزندآوری و همچنین از بین بردن عواملی که مانع فرزندآوری می شوند پایه ای برای آموزش و راهکاری مناسب بمنظور قدم برداشتن در راستای سیاستهای جدید جمعیتی کشور و ترغیب زوجین به فرزندآوری بودند. نویسنده اول :قدرت اله شاکری نژادعضو هیئت علمی گروه پژوهشی آموزش سلامت، جهاد دانشگاهی خوزستان نویسنده مسئول :مریم ساکی عضو هیئت علمی گروه پژوهشی آموزش سلامت، جهاد دانشگاهی خوزستان فرزانه جاروندی: کارشناس پژوهشی گروه پژوهشی آموزش سلامت جهاد دانشگاهی خوزستان سعیده حاجی نجف: کارشناس علوم تغذیه ، کارشناس گروه پژوهشی آموزش سلامت

Title: بررسی سطح هوش سازمانی، یادگیری سازمانی و چابکی سازمانی در بیمارستان های آموزشی تحت پوشش دانشگاه علوم پزشکی یزد

http://tbj.ssu.ac.ir/article-1-1886-fa.html

مقدمه: هوش سازمانی عبارت است از استعداد و  ظرفیت یک سازمان در جهت دهی به توانایی  های ذهنی و استفاده از این توانایی ها برای رسیدن به رسالت سازمانی ، یادگیری سازمانی به معنای فرآیند کشف و اصلاح اشتباهات و چابکی سازمانی به معنای توانایی واکنش سریع به تغییرات محیطی و عاملی برای اثر بخشی بیمارستان به حساب می آید. هدف از انجام این مطالعه بررسی وضعیت هوش سازمانی ، یادگیری سازمانی و چابکی سازمانی در بیمارستان های آموزشی تحت پوشش دانشگاه علوم پزشکی یزد بود. روش بررسی: مطالعه حاضر تحلیلی و از نوع مقطعی بود که در سال 1394صورت پذیرفت. جامعه پژوهش شامل کادر اداری و درمانی بیمارستان های شهید صدوقی، افشار، رهنمون و سوانح سوختگی بود . 370 نفر از کادر درمانی و اداری که بصورت نمونه گیری طبقه بندی با تخصیص متناسب انتخاب شدند در مطالعه  شرکت کردند. داده ها با استفاده از پرسشنامه های هوش سازمانی آلبرخت (2002) ، یادگیری سازمانی نیفه(2001) و چابکی سازمانی  برگرفته از نظریه شریفی و ژانگ( 1999)جمع آوری گردید.  با استفاده از آمار توصیفی و تحلیلی بوسیله نرم افزار   SPSS18 تجزیه و تحلیل شد. یافته ها: میانگین نمره هوش سازمانی  بیمارستان ها 29/2 ، میانگین نمره یادگیری سازمانی بیمارستان ها 48/1و میانگین نمره چابکی سازمانی بیمارستان ها 52/1 بود . همچنین متغیر بیمارستان و تحصیلات بر هوش سازمانی ، یادگیری سازمانی و چابکی سازمانی اثر می گذارند. نتیجه گیری: یافته های مطالعه نشان می دهند که به منظور ارتقای هوش سازمانی ، یادگیری سازمانی و چابکی سازمانی بیمارستان ها، استفاده از استراتژی ها و راهکارهایی چون توسعه حرفه ای کارکنان ، ساختار سازمانی منعطف و آمادگی برای مواجهه با تغییرات محیطی لازم است .

Title: تأثیر آموزش هوش معنوی بر بهبود کیفیت زندگی معلولان جسمی شهرستان میبد

http://tbj.ssu.ac.ir/article-1-1250-fa.html

چکیده مقدمه: کیفیت زندگی معلولان جسمی موضوع مهمّی است که پژوهش حاضر به منظور پرداختن به این موضوع، به بررسی تأثیر آموزش هوش ‌معنوی بر بهبود کیفیّت ‌زندگی معلولان جسمی بزرگسال زن و مرد شهرستان میبد در سال 1393اقدام کرده است. روش بررسی: در این پژوهش که از نوع مطالعات شبه آزمایشی- مداخله‌ای و به صورت پیش‌آزمون- پس‌آزمون با گروه کنترل بود، 28 نفر زن و مرد معلول شرکت داشتند (7 مرد و 21 زن) که به صورت نمونه‌گیری هدفمند انتخاب شده و در دو گروه آزمایش و گواه قرار گرفتند. بعد از اجرای پیش‌آزمون، دو گروه از نظر کیفیت زندگی به وسیله‌ی آزمون  tدو نمونه مستقل مقایسه شدند و بعد از اطمینان از عدم تفاوت معنادار بین دو گروه، 10جلسه 90 دقیقه‌ای آموزش هوش ‌معنوی برای گروه آزمایش اجرا شد و گروه گواه آموزشی دریافت نکردند. برای هر دو گروه بعد از جلسات آموزش، پس آزمون کیفیّت ‌زندگی SF-36 اجرا شد . تجزیه و تحلیل داده‌ها با روش آماری تحلیل کواریانس یک‌راهه انجام شد. یافته ها: نتایج نشان داد که گروه آزمایش نسبت به گروه گواه از نظر کیفیّت‌ زندگی ‌کلّی، سلامت ‌عمومی، محدودیّت‌ ایفای نقش به دلایل جسمانی، محدودیّت ایفای نقش به دلایل عاطفی، عملکرد ‌اجتماعی و نشاط، به طور معناداری نمره‌ی بالاتری کسب کردند.  افزایش نمره‌ی شرکت کنندگان در کلاس‌ها فقط در عملکرد جسمانی، درد بدنی و سلامت روان معنادار نبود. نتیجه گیری: با توجّه به یافته‌ها می‌توان نتیجه گرفت آموزش هوش ‌معنوی می‌تواند باعث بهبود کیفیّت‌ زندگی معلولان شود.

Title: ارزیابی عملکرد پایگاه های اورژانس پیش بیمارستانی سطح شهر یزد بر اساس مدت زمان لازم جهت حضور بر بالین بیماردر سال1392

http://tbj.ssu.ac.ir/article-1-1103-fa.html

مقدمه : یکی از اجزاء ضروری و سرنوشت ساز در در مان بیماران بدحال، خدمات پزشکی اورژانس می باشند. اصلی مشترک در خدمات اورژانس پیش بیمارستانی وجود دارد که بصورت یک هدف در آمده است و آن عبارت است زمان پاسخ 8 دقیقه یا کمتر جهت حمایت پیشرفته از زندگی افراد در حوادث تهدید کننده حیات. لذا این پژوهش با هدف ارزیابی عملکرد اورژانس پیش بیمارستانی شهر یزد بر اساس مدت زمان لازم جهت حضور بر بالین بیمار در سال 92 انجام شده است. روش بررسی: پژوهش حاضر توصیفی تحلیلی است که بصورت مقطعی انجام شده است. روش جمع آوری داده ها بصورت سرشماری و روش تحلیل داده ها بصورت کیفی بوده است به این صورت که بر اساس روش لیکرت عملکرد قبل از 8 دقیقه به عنوان عملکرد عالی تا 8دقیقه عملکرد خوب و بالاتر از 8 دقیقه عملکرد ضعیف در نظر گرفته شده است. یافته ها: طبق داده های بدست آمده از پژوهش، در طول سا ل 92 به تفکیک پایگاههای سطح شهر یزد بهترین میانگین عملکرد زمانی مربوط به پایگاه منطقه 4 با 58/8دقیقه و ضعیف ترین عملکرد مربوط به پایگاه منطقه 6 با 01/12 دقیقه بوده است. عملکرد ماموریتهای شهری مرکز مدیریت حواث و فوریتهای پزشکی استان یزد بطور کلی در سال 92 عبارت بود از: 84/9 دقیقه. یافته ها نشان دادند که بالاترین میانگین عملکرد زمانی مربوط به فصل زمستان با 2/10 دقیقه و کمترین میانگین زمان عملکردی مربوط به فصل تابستان با 47/9 دقیقه بوده است. در ادامه نتایج چنین نشان دادند با اینکه تعداد ماموریتها در فصل تابستان نسبت به زمستان بیشتر بوده است،  7/25% در مقابل 1/24%، ولی میزان فوتی ها در این فصل کمتر از فصل زمستان بوده است ( 31/22% در مقابل 54/27% ). نتیجه گیری: در مقایسه اطلاعات بدست آمده حاصل از یافته های پژوهش، به تاثیر مدت زمان ماموریت در کاهش میزان مرگ و میر پی برده شد لذا با توجه به حساسیت فعالیت های اورژانس های پیش بیمارستانی لزوم توجه به بازه زمانی ماموریتهای شهری مرکز مدیریت حوادث و فوریتهای پزشکی استان یزد آشکار گردید که این مهم جز با توجه و نظارت مسئولین مربوطه و تهیه شرایط و امکانات کافی مقدور نمی باشد.

Title: بررسی میزان انتقال بین مراحل بیماری قلبی و ارتباط سندروم متابولیک با استفاده از مدل مارکف

http://tbj.ssu.ac.ir/article-1-1335-fa.html

چکیده مقدمه: درحال حاضر بیماری های قلبی وعروقی علّت اصلی مرگ در جهان است، در ایران نیزسکته قلبی حاد یکی از علل اصلی مرگ و میر می باشد. هدف از این مطالعه، تعیین نرخ انتقال بین وضعیتهای بیماری قلبی ومطالعه اثرمخاطره ی سندرم متابولیک براین انتقالها با استفاده از برازش مدل چند وضعیتی مارکف می باشد. روش بررسی: با پیگیری 5 ساله بیماران مبتلا به انفارکتوس حاد میوکارد (با و بدون بالا رفتن قطعه ی ST) بستری در بخش مراقبت های ویژه بیمارستان های شهر یزد در سال های1380 و  1381 که اطّلاعات مربوط به آنها ثبت  شده بود.457 نمونه به روش نمونه گیری آسان از میان بیماران قلبی مراجعه کننده انتخاب شدند. یافته ها: طی پنج سال یک فرد بیمار که در حال حاضر در وضعیت نرمال می باشد  با احتمال 26% دچار علائمی نظیر درد قفسه ی سینه خواهد شد، با احتمال 11% وارد مرحله ی حادی از بیماری خود می شود و با احتمال 24% خواهد مرد.سندرم متابولیک با 0598/1=HR  بیانگر این است که این متغیر خطر شروع مجدد بیماری قلبی را 6% و خطر مرگ را برای فردی که در وضعیت نرمال می باشد به میزان 38% افزایش می دهد (3778/1HR=). نتیجه گیری: وقتی چند پیامد وجود دارد استفاده از مدل های چند وضعیتی مارکف مناسب است. برآورد نرخ انتقال ،مطالعه اثرعوامل مخاطره براین انتقالات و بررسی اثرات مداخلات پزشکی در صورت وجود از مواردی هستند که برآورد آنها در مطالعات مرتبط با تصمیم گیری های پزشکی حایز اهمیت هستند.   این مقاله بر گرفته از پایان نامه دانشجوی کارشناسی ارشد آمارزیستی،دانشگاه علوم پزشکی و خدمات بهداشتی درمانی شهید صدوقی یزد می باشد.

Title: بررسی نگرش مراجعین به مراکز مختلف درمانی دندانپزشکی شهر یزد در رابطه با باورهای نادرست دندانپزشکی

http://tbj.ssu.ac.ir/article-1-2409-fa.html

مقدمه: نقش باورهای نادرست به عنوان مشکل شناخته شده‌ای در جلوگیری از دستیابی به سطح مطلوبی از بهداشت دهان و دندان در جامعه انکارناپذیر است. مطالعه حاضر با هدف بررسی نگرش مراجعین به مراکز مختلف درمانی دندانپزشکی شهر یزد در رابطه با باورهای نادرست دندانپزشکی در سال 1391 انجام شد. روش بررسی: در این مطالعه توصیفی- مقطعی،300 نفر از مراجعین به مراکز مختلف درمانی دندانپزشکی شهر یزد به روش نمونه‌گیری ‌خوشه‌ای ‌تصادفی ‌دو‌ مرحله‌ای انتخاب شدند. داده‌ها با استفاده از پرسشنامه‌ای روا و پایا که شامل دو بخش اطلاعات جمعیت‌شناختی و باورهای مردم در رابطه با دندانپزشکی بود، جمع‌آوری گردید، سپس نمره نگرش افراد به سه گروه ضعیف(99/1-1)، متوسط (99/3-2) و قوی (5-4) تقسیم شد. داده‌ها با استفاده از نرم افزار آماری ‌ SPSSنسخه 16 و آزمون‌‌‌های آماری,Tukey  ,ANOVA T-test و ضریب همبستگی پیرسون تجزیه و تحلیل شد. نتایج: میانگین نمره نگرش در‌ جامعه مورد بررسی63/0±87/2 بود. 50% از مراجعین، ‌‌نگرش متوسطی نسبت به خدمات دندانپزشکی داشتند و تنها ‌‌7‌/14% دارای نگرش در سطح بالا بودند. میانگین اعتقاد به باورهای نادرست، در زنان و مردان اختلاف ‌آماری معنی‌داری نداشت(363/0p=). اما رابطه معنی داری بین سن، میزان تحصیلات و شغل افراد، با میانگین اعتقاد به باورهای نادرست وجود داشت(001/0p=) نتیجه‌گیری: میزان باورهای نادرست در مورد دندانپزشکی در ‌جامعه مورد بررسی از نظر شدت در سطح متوسط بوده و از نظر وسعت در نیمی از افراد جامعه وجود داشت. لذا به منظور دستیابی به نتایج مطلوب در زمینه بهداشت دهان، برنامه‌ریزی به منظور افزایش سطح آگاهی افراد، قبل از هر گونه برنامه ریزی جهت اصلاح سطح سلامت دهان و دندان ضروری است.

Title: ارزیابی اثر روغن فرار میخک بر شاخص‌ اکسیداسیون و تجمع زیستی (کبد و عضلات) به دنبال مصرف سرب در بلدرچین ژاپنی

http://tbj.ssu.ac.ir/article-1-1846-fa.html

مقدمه: برخی فلزات سنگین در غلظت­های پایین سمی هستند و می­توانند رادیکال آزاد ایجاد کنند که در نهایت منجر به آسیب سلولی شوند. روغن­های فرار دارای خواص آنتی اکسیدانی هستند و توانایی جذب و به دام­اندازی برخی عناصر سمی را دارند، در نتیجه مانع تجمع بافتی این فلزات می شوند. روش بررسی: یک مطالعه تجربی روی 280 بلدرچین در گروه­های مختلف انجام شد.برای تست سنجش میزان اکسیداسیون چربی­های بافتی در کبد و عضلات بلدرچین از تست (TBARS) و جهت تعیین میزان تجمع بافتی سرب از دستگاهICP-OES استفاده گردید. آنالیز دادهها توسط آزمون one-way ANOVA و تست تکمیلی Duncan با استفاده از نرم افزار SPSS نسخه 20 و سطح معنی­داری 05/0 P<انجام شد. یافته ها: نتایج تست تیبارس در سنین 21 و 35 روزگی نشان داد که میانگین غلظت مالون دی آلدهید کبد و عضلات در گروه دریافت کننده سرب به طور معنی­داری (05/0>p)بالاتر از سایر گروه­ها بود.نتایج حاصل از اندازه­گیری سرب در سن 21 روزگی نشان داد که در این سن تجمع سرب در کبد و عضلات زیر حد تشخیصی دستگاه است.در سن 35 روزگی میانگین میزان تجمع سرب در بافت کبد و عضلات در گروه­های دریافت کننده سرب به همراه 450ppm  روغن فرار میخک، 100 ppm  روغن فرار میخک یا 500 ppm  ویتامین C به طور معنی­داری نسبت به گروه دریافت کننده سرب پایین­تر بود(05/0>p). نتیجه گیری: نتایج نشان داد که روغن فرار میخک در کاهش میزان غلظت مالون دی آلدهید و کاهش میزان تجمع سرب در بافت کبد و عضلات مؤثر بود.

Title: تأثیر مسئولیت اجتماعی بر وفاداری مشتری با توجه به نقش واسطه‌ای تصویر ذهنی و اعتماد بیماران به بیمارستان‌های آموزشی شهر یزد

http://tbj.ssu.ac.ir/article-1-1847-fa.html

مقدمه: یکی از فاکتورهای عمده موفقیت سازمان ها، وفاداری مشتریانشان است. ازاین‌رو بایستی عوامل مؤثر بر این سازه توسط کلیه سازمان‌های تولیدی و خدماتی موردبررسی قرارگرفته و در دستور کارمدیران آن سازمان قرار گیرد. ازاین‌رو در این تحقیق به بررسی تأثیر مسئولیت اجتماعی بیمارستان‌های آموزشی شهر یزد بر وفاداری بیماران با توجه به نقش واسطه‌ای اعتماد وتصویرذهنی بیماران از آن بیمارستان پرداخته‌شده است. روش بررسی: این تحقیق از نظر هدف کاربردی و از نظر شیوه گردآوری اطلاعات تحقیق توصیفی-پیمایشی و از حیث ارتباط بین متغیرها از نوع همبستگی- علی می­باشد. ابزار جمع‌آوری اطلاعات در این تحقیق پرسشنامه می‌باشد که با توجه به حجم نمونه موردنظر بین 384 نفر توزیع شد. همچنین در آزمون اولیه پرسشنامه ضریب آلفای کرونباخ محاسبه‌شده برای سازه‌های پژوهش بالاتر از 7/0 کسب گردید.درنهایت جهت تجزیه‌وتحلیل داده‌های تحقیق از روش همبستگی پیرسون برای بررسی رابطه بین متغیرها و از روش تحلیل مسیر جهت بررسی روابط علی آن‌ها در فضای Spss و Amos استفاده گردید. یافته‌ها: نتایج تحقیق در بخش آمار توصیفی نشان می‌دهد که بیشترین پاسخگویان از قشر کارمند و مرد و بالای 45 درصد آن‌ها دررده سنی50-41 سال بوده‌اند و در بخش آمار همبستگی نشان می‌دهد که کلیه روابط مدل تحقیق با 99 درصد اطمینان دارای رابطه معناداری هستند. نتیجه‌گیری: نتایج تحقیق نشان‌داد که مسئولیت اجتماعی بیمارستان می‌تواند تأثیر مثبتی بروفاداری بیماران نسبت به بیمارستان بگذارد، چرا که با افزایش میزان مسئولیت اجتماعی، تصویر ذهنی خوبی از بیمارستان و خدماتی که ارائه می دهد در ذهن بیماران ایجاد گردیده و منجر به افزایش اعتماد آنها خواهد شد. البته با توجه به اینکه در این مطالعه بیمارستان های آموزشی و کم هزینه برای مطالعه انتخاب گردیدند، مسئولیت اجتماعی بر اعتماد بیماران به صورت مستقیم تأثیری نداشته است.

Title: ارزیابی خصوصیات عملکردی و حسی خامه مخلوط گیاهی و لبنی

http://tbj.ssu.ac.ir/article-1-2410-fa.html

چکیده مقدمه: امولسیون های روغن در آب به دلیل ویژگی های فیزیکی خود توجه چشمگیری را در صنایع غذایی به خود معطوف کرده اند. اما وجود میزان قابل ملاحظه ای از کلسترول در برخی از آنها نظیر خامه، یک عامل خطرزا در شیوع بیماری های قلبی عروقی بوده و در اثر اکسیداسیون کلسترول در طی فرایند یا نگهداری مواد غذایی، ترکیباتی ایجاد می شوند که بعنوان عوامل سرطانزا، سیتوتوکسیک، موتاژنیک و آتروژنیک شناخته شده اند.  هدف از این تحقیق، تولید خامه مخلوط با کلسترول و قیمت کمتر و ارزش تغذیه ای بیشتر نسبت به خامه لبنی است. روش بررسی: این پژوهش یک مطالعه تجربی بود که در آن سه فرمولاسیون مختلف از خامه مخلوط(A,B,C) با استفاده از شیر سویا، شیر گاو، امولسیفایر محلول در آب، زانتان، کلرید کلسیم، خامه سبک لبنی، روغن آفتابگردان و امولسیفایر سیتروم تهیه شد. از همبستگی پیرسون جهت ارزیابی رابطه میان اورران و آب انداختگی با میزان چربی و پروتئین استفاده شد. ارزیابی حسی نمونه های خامه مخلوط با استفاده از مقیاس 9 نقطه ای هدونیک انجام شد. با توجه به اینکه متغیرهای پاسخ رتبه ای بودند از آزمون ناپارامتری کروسکال والیس استفاده شد. برای مقایسه دو به دو گروه ها از آزمون اختصاصی مقایسه های چندگانه دان و همچنین برای مقایسه ویژگی های کمی از آمار توصیفی بصورت میانگین و انحراف معیار استفاده گردید و تجزیه و تحلیل داده ها با نرم افزار R 2.3 انجام گرفت. یافته ها: با استفاده از نرم افزار minitab.16 ضریب همبستگی پیرسون معنی داری میان چربی و اورران(افزایش حجم) برابر 45/0 و03/0 = p و میان اورران و آب انداختگی برابر 87/0-  و001/0 = p  بدست آمد. بیشترین امتیاز طعم، رنگ، حالت خامه ای و پذیرش کلی، به ترتیب برای فرمولاسیون های C و بیشترین امتیاز بافت برای فرمولاسیون C>A>B  بدست آمد ( 05/0≤  p). نتیجه گیری: نتایج حاصل از این مطالعه نشان داد که استفاده از شیر سویای تهیه شده از آرد کامل سویا گرچه تا حدودی طعم و آب انداختگی خامه مخلوط را تحت تأثیر قرار می دهد ولی ضمن افزایش خواص تغذیه ای، می تواند در کنار سایر اجزای فرمولاسیون، بافت مناسبی را ایجاد کند.
